# Sick Soldiers and Their Nurses

**Published:** 1887-10-15

**Authors:** 


### Sick Soldiers and their Nurses.

Britons are a warlike race, and the sight of the red coats with the sounding of the trumpets always rouses the martial spirit in the tamest British bosom. "The pomp and circumstance of glorious war" have fired the heart of many a youth in all ranks and filled him with enthusiasm for the gallant march and the heroic field. When men get a little older their ardour cools, and probably at thirty they would not be so ready to choose the military career as they were at eighteen. But having chosen it they are minded, like sturdy sons of Britain, to stick to their trade and do their duty like men. They do their duty like men in many a strange and far-off corner of the earth, on a foreign shore, under a foreign sky, where neither father, mother, sister, nor friend can help them when they are sick, or close their eyes if the fortunes of war should bring them to an early death. The young soldier, be he officer or be he private, has often a wonderful kind of heroism about him which those at home know little of. He may not be frequently called upon to march to the field of danger, which to some is also a field of death. But he is constantly ordered to strange lands, under new, untried, and unhealthy circumstances, where he contracts dysentery, cholera, typhoid fever, ague, and all manner of painful, weakening, depressing, and dangerous illnesses. Any one of these diseases is serious and alarming, even at home, where, added to the highest medical ability, is the most skilled and perfect nursing and the reassuring presence of near relatives and friends. But the poor soldier in India and elsewhere must endure the pains and tortures of burning fever, the sleepless nights, the tremendous feelings of depression and impending death, not only without the sight of friends or the touch of a mother's or sister's hand, but without even a nurse, except the male orderly who may be told off to watch him and render him such service as his experience or inexperience may suggest. No doubt some of these men nurses are kind, brotherly fellows, and do everything which a man's heart can prompt. But a man is not like a woman, and never can be; and when we want a nurse let us have wife, or mother, or sister, or at least a gentle friend who feels as mother, or sister, or tender wife would feel.

Many people at home are under the impression that our soldiers have every possible attention and care in the way of skilled lady nursing; and so they ought to have, but they have not. What is everybody's business is nobody's business —the more ashamed should we all be that we have never made it our own business. If men give up their whole prospects in life to fight our battles, or at least to be always ready to fight them, the very least we can do in return is not only to say "Hip, hip, hurrah" when they return from the war, but to make certain that they are amply provided with every possible degree of comfort, skilled nursing, and medical attendance when they are wounded or disabled from any other cause. Personally we feel ashamed that at this period of our national history anything should yet remain to be done in this direction.

However, something has been done, and that within the last few years. Thanks to Lady Roberts, the noble wife of a brave and honourable husband, a scheme has been matured for the employment of lady nurses in British military hospitals in India, and the Government have not only sanctioned the movement and undertaken the whole costs in connection with it, but have decided that a commencement shall at once be made at the two large military centres of Umballa and Rawalpindi. So far, good. But more requires to be done. Europeans, and especially young lady-nurses of the class which it is proposed to send out, break down rapidly under the combined strain of their anxious work and the fierce heat of the new and untried climate. If these ladies are to do the good which they are fitted to accomplish, and to retain the health and strength which their devotion and our honour and gratitude alike demand that they shall retain, they must have "Homes in the Hills" provided, to which they may from time to time resort as their failing strength warns them of the approach of an impending breakdown. Wanting such homes, there is nothing for it but a short period of service; then broken health; and finally a return to this country, disabled, disheartened, and permanently enfeebled. Have we any right—would any of us wish to ask young and devoted women to undertake these duties at such risk and cost to themselves? If they are prepared to go to India we should and must gladly do all we can to make their going not only as little dangerous as possible, but as prosperous and happy as the nature of their arduous duties will permit.

The Government of India do not intend to provide the "Homes in the Hills" which the promoters of the scheme so ardently desire, and declare to be so essential to permanent success. An appeal is therefore to be made to the general public, as it has already been made to military circles. No doubt soldiers and others at home will largely respond to such an appeal; but with whatever force it may be urged upon military circles, it should be felt to come with tenfold urgency to the hearts of those who have given neither themselves, nor their children, nor their relatives, but only their money, in the great cause of their country's honour and defence. We do not desire to multiply words; nor do we think they are necessary. If our sick and wounded soldiers have inspired young and delicately nurtured ladies to give themselves up regardless of risk to the laborious, anxious, and dangerous duties of nursing them, the smallest we can think of is to make it certain that when these ladies in their turn are disabled by sickness and toil, they too shall be taken to a cheerful and healthy home, where their strength may be recruited and their spirits reanimated for fresh duties. Whoever has a patriotic and generous heart will not wait to be asked twice in so noble a cause. A bazaar is being organised on a large scale at Portsmouth in aid of Lady Roberts' Fund, and any contributions of money, materials, articles for sale, or work will be gratefully received and acknowledged in THE HOSPITAL if sent direct to Mrs. Crease, Eastney Barracks, Portsmouth. Those who desire more information on the subject, or who may wish to offer immediate help, should apply to Mrs. Crease.

